# Undisturbed tubal pregnancies with positive fetal heart treated medically: Case study

**DOI:** 10.34763/jmotherandchild.20222601.d-22-00037

**Published:** 2023-02-22

**Authors:** Mariam Obaid, Mohannad Abu-Faza, Ibrahim A. Abdelazim, Hanan S. Al-Khatlan, Aliaa M. Al-Tuhoo

**Affiliations:** Department of Obstetrics and Gynecology, Ahmadi hospital, Kuwait Oil Company (KOC), Ahmadi, Kuwait; Department of Obstetrics and Gynecology, Faculty of Medicine, Ain Shams University, Cairo, Egypt

**Keywords:** Tubal, Pregnancies, Fetal, Heart, Methotrexate

## Abstract

The incidence of ectopic pregnancy (EP) is 1.3-2.4%. Suspicion of EP starts after a positive serum pregnancy test and failure to visualize the intrauterine gestational sac (GS) by transvaginal sonography (TVS). About 88% of tubal EPs are diagnosed by absent intrauterine GS and the presence of an adnexal mass during TVS. Medical treatment of EP using methotrexate (MTX) is cost-effective with a similar success rate to surgical treatment. The presence of fetal heart beats, β-human chorionic gonadotropin >5000 mIU/mL, and EP size >4 cm are relative contraindications for using MTX in the treatment of EP.

## Introduction

The rising incidence of ectopic pregnancies (EPs) can be explained by the increased ARTs rate, tubal surgeries, and improved diagnostic techniques [1].

The incidence of EP is 1.3-2.4%, and a ruptured EP is a direct cause of maternal death in the first trimester of pregnancy [2]. The oocyte fertilization occurs in the fallopian tube (FT). The migration of fertilized oocytes to the uterus is facilitated by FT cilia and muscles. FT dysfunction and/or inflammation is implicated in oocyte retention and subsequent EP [3].

Prior tubal surgery, sterilization, prior EP, and intrauterine contraceptive device (IUCD) were considered high-risk factors for EP. Infertility, prior pelvic inflammatory disease (PID), smoking, and multiple partners were considered moderate risk factors for EP [4].

This report highlights that the presence of fetal heart activity is one of the relative contraindications of using methotrexate (MTX) in the treatment of undisturbed tubal EPs.

### Case-Series

Two cases of undisturbed tubal-EPs from Ahmadi Hospital, Kuwait were included in this report after departmental approval and written consent to publish their data as a case study.

Inclusion criteria included women with initial β-human chorionic gonadotropin (β-hCG) <5000 mIU/mL, EP size <4 cm with positive fetal heart activity, those who refused the option of laparoscopic surgery, and those who were reliable for follow-up. **Case 1**: A 35-year-old woman, P3 (all by cesarean sections), 7 gestational weeks^+2days^, was admitted with iliac fossa pain, after a positive pregnancy test, as a case of pregnancy of unknown location (PUL) when the trans-vaginal ultrasound (TVS) showed an empty uterus at β-hCG level 3614 mIU/mL.

The departmental ultrasound, according to the hospital`s protocol, showed an empty uterus, with a left adnexal echogenic gestational sac (GS) (38 X 32 mm) with a fetal pole inside (bagel sign), and positive fetal heart activity. The color Doppler examination showed a ring-of-fire sign around the GS, indicating exaggerated blood flow around the GS.

She was diagnosed as an undisturbed tubal EP with positive fetal heart activity. She refused the option of laparoscopic surgery. Therefore, she was counselled for medical treatment using methotrexate (MTX). She was also informed of MTX’s side effects, failure rate, repeated MTX dose(s), serial β-hCG assay, and the long hospital stay for observation.

After written consent, she received the first 75 mg MTX dose (50 mg/BSA) at 3614 IU/mL β-hCG level. The 4^th^ day β-hCG after the first MTX dose increased to 5421 mIU/mL (TVS showed negative fetal heart beats), and the 7^th^ day β-hCG was 5055 mIU/mL [<15% decrease of β-hCG (6.75%)]. Following this, the second 75 mg MTX dose was administered. The 4^th^ day β-hCG after the second MTX dose was 3851 mIU/mL, and the 7^th^ day β-hCG was 2218 mIU/ml [>15% decrease of β-hCG (42.4%)] [Table 1].

**Table 1:** The serial β-hCG levels of studied cases

β-hCG (mIU/ml)	Case 1 (Received two MTX-doses)	Case 2 (Received one MTX-dose)
Initial β-hCG (mIU/ml)	3614	1608
4^th^ day β-hCG (mIU/ml) after the 1^st^ MTX-dose	5421	1831
7^th^ day β-hCG (mIU/ml) after the 1^st^ MTX-dose	5055	1325
4^th^ day β-hCG after the 2^nd^ MTX-dose	3851	-
7^th^ day β-hCG after the 2^nd^ MTX-dose	2218	-
2^nd^ week β-hCG	292.4	595.1
3^rd^ week β-hCG	77.84	151.1
4^th^ weeks β-hCG	18.58	73.1
5^th^ week β-hCG	-	32.1
6^th^ week β-hCG	-	10.09
7^th^ week β-hCG	0.100	3.42

hCG: Human chorionic gonadotropin. MTX: Methotrexate. Normal β-hCG value 0.0-10 mIU/mL

She was discharged home for follow-up in OPD after satisfactory TVS findings (GS decreased to 18 X 14 mm). The 2^nd^ week β-hCG after the second MTX dose was 292.4 mIU/mL, and the 3^rd^ week β-hCG was 77.84 mIU/mL, while the 4^th^ week β-hCG was 18.58 mIU/mL (GS completely disappeared by TVS), and the 7^th^ week β-hCG was 0.100 mIU/mL [Table 1]. She did not attend the follow-up appointments for the 5^th^ and 6^th^ weeks.

**Case 2**: A 30-year-old woman, primipara, 6 weeks’ gestation, was referred to our hospital as a case of undisturbed tubal EP with positive fetal heart activity when she refused the option of laparoscopic surgery.

The departmental ultrasound at initial β-hCG 1608 mIU/mL showed an empty uterus, with an adnexal gestational sac (GS) (32 X 28 mm) with a fetal pole inside (bagel sign), and positive fetal heart activity.

The studied woman was counselled for medical MTX treatment. She was informed of MTX’s side effects, failure rate, repeated MTX dose(s), serial β-hCG assay, and the long hospital stay for observation.

After written consent, she received the first 50 mg MTX dose (50 mg/BSA) at 1608 IU/mL β-hCG level [Table 1].

The 4^th^ day β-hCG after the first MTX dose increased to 1831 mIU/mL (TVS showed negative fetal cardiac activity), while the 7^th^ day β-hCG was 1325 mIU/mL [>15% drop of β-hCG (27.6%)]. Therefore, she was discharged home for follow-up in OPD after satisfactory TVS findings (GS decreased to 14 X 12 mm).

The 2^nd^ week β-hCG was 595.1 mIU/mL, while the 3^rd^ week β-hCG was 151.1 mIU/mL, and the 4^th^ week β-hCG was 73.1 mIU/mL (GS completely disappeared by TVS). The 5^th^ week β-hCG was 32.1 mIU/mL, the 6^th^ week β-hCG was 10.09 mIU/ mL, and the 7^th^ week β-hCG was 3.42 mIU/mL [Table 1].

## Discussion

Suspicion of EP starts after positive serum pregnancy test and failure to visualize intrauterine GS by TVS (PUL) [2,5].

The PUL diagnosis changed after visualization of intrauterine GS to intrauterine pregnancy (IUP) in 30% of cases, while in 70% of cases it changed to either miscarriages or EP [2].

A retrospective review of PUL suggests ≥35% β-hCG rise in 48 hours to diagnose IUP [6], while <35% β-hCG rise in 48-hours has an 80.2% overall accuracy in diagnosing EP [2]. Identification of an adnexal echogenic structure (GS) with yolk sac ***(bagel sign)*** and Doppler blood flow around ***(ring of fire)*** without intrauterine GS is highly suggestive for EP [2].

A systematic review found that 88% of tubal-EPs were diagnosed by absent intrauterine GS and presence of an adnexal mass during TVS [7].

Medical MTX treatment of EP is cost-effective with a similar success rate to surgical treatment [8].

The presence of fetal heart beats, β-hCG >5000 mIU/mL, EP size >4 cm, or unreliable patients are relative contraindications for using MTX in the treatment of EP [2].

The β-hCG should be checked on days 4 and 7 following MTX treatment of EP. A systematic review showed that the success rate with single MTX dose was 81.1% when the initial β-hCG levels was between 10.000 and 15.000 mIU/mL [9].

The two doses of MTX were suggested by Barnhart et al. with a 87% success rate [10]. Decreased β-hCG by ≥15% on day 7 indicate successful MTX treatment, and the β-hCG should be monitored weekly till it reaches the non-pregnant level [2].

Regardless of which MTX regimen is used, if the β-hCG does not decrease adequately (<15% on day 7) after MTX or if it is increased, another MTX dose or surgical options should be considered [4].

The first studied case (Case 1) received two doses of MTX because the β-hCG decreased by <15% (6.75%) on day 7 after the first MTX dose (from 5421 to 5055 mIU/mL on day 4 and 7, respectively). The second MTX dose was successful and the β-hCG decreased by >15% (42.4%) on day 7 (from 3851 to 2218 mIU/ml on day 4 and 7, respectively).

The second studied case (Case 2) received one dose of MTX because her initial β-hCG was low (1608 mIU/mL) compared to the first studied case. The 4^th^ day β-hCG after the first MTX dose increased to 1831 mIU/mL, while the 7^th^ day β-hCG was 1325 mIU/mL [>15% drop of β-hCG (27.6%)].

After MTX, usually the β-hCG returns to the non-pregnant level within 2-3 weeks, but it can take up to 8 weeks in EPs with higher initial β-hCG [2,8].

## Conclusion

The presence of fetal heart activity is one of the relative contraindications (not absolute contraindication) of using MTX in the treatment of undisturbed tubal EPs. MTX can be used for medical treatment of undisturbed tubal EPs with positive fetal heart activity in special situations (i.e. refusal of surgery) after proper counselling.

## References

[1] Taran FA, Kagan KO, Hübner M, Hoopmann M, Wallwiener D, Brucker S (2015). The diagnosis and treatment of ectopic pregnancy. Dtsch Arztebl Int.

[2] Abdelazim I, AbuFaza M, Shikanova S, Karimova B (2021). Diagnostic Criteria and Treatment Modalities of Ectopic Pregnancies: A Literature Review. EMJ Repro Health.

[3] Abdelazim IA, Shikanova S, Zhurabekova G, Kanshaiym S, Karimova B, Sarsembayev M (2019). Open cornual resection versus laparoscopic cornual resection in patients with interstitial ectopic pregnancies. Eur J Obstet Gynecol Reprod Biol.

[4] Obaid M, Abdelazim IA, Abu-Faza M, Rajendran S, Elhaddad SA (2022). Treatment of left tubal pregnancy with foetal cardiac activity using a two-dose methotrexate regimen. Menopause Review/Przegląd Menopauzalny.

[5] Abdelazim IA, Nussair B, Zhurabekova G, Svetlana S, Abu-Faza M, Naser W (2018). Comment on an intrauterine gestational sac surrounded by thin myometrium at fundus. J Med Ultrasound.

[6] Morse CB, Sammel MD, Shaunik A, Allen-Taylor L, ­Oberfoell NL, Takacs P (2012). Performance of human chorionic gonadotropin curves in women at risk for ectopic pregnancy: exceptions to the rules. Fertil Steril.

[7] Crochet JR, Bastian LA, Chireau MV (2013). Does this woman have an ectopic pregnancy? the rational clinical examination systematic review. JAMA.

[8] Abdelazim IA, Abu-Faza M, Zhurabekova G, Shikanova S, Kanshaiym S, Karimova B (2019). Successful pregnancy outcome immediately after methotrexate treatment for cesarean section scar pregnancy. Gynecol Minim Invasive Ther.

[9] Menon S, Colins J, Barnhart KT (2007). Establishing a human chorionic gonadotropin cutoff to guide methotrexate treatment of ectopic pregnancy: a systematic review. Fertil Steril.

[10] Barnhart KT, Hummel AC, Sammel MD, Menon S, Jain J, Chakhtoura N (2007). Use of “2 dose” regimen of methotrexate to treat ectopic pregnancy. Fertil Steril.

